# Cross-cultural adaptation and validation of the Simplified Chinese version of Copenhagen Hip and Groin Outcome Score (HAGOS) for total hip arthroplasty

**DOI:** 10.1186/s13018-018-0971-2

**Published:** 2018-11-06

**Authors:** Shiqi Cao, Jia Cao, Sirui Li, Wei Wang, Qirong Qian, Yu Ding

**Affiliations:** 1 Department of Rehabilitation, Minimally Invasive Spine Center, Navy General Hospital, No. 6, Fucheng Road, Haidian District, Beijing, 100048 People’s Republic of China; 2 Joint Surgery and Sports Medicine Department, Changzheng Hospital, Navy Medical University, No. 415, Fengyang Road, Huangpu District, Shanghai, 200003 People’s Republic of China; 3 College of Basic Medicine, Army Medical University, Chongqing, 400038 People’s Republic of China; 4 Department of Orthopaedics, Chengdu Military General Hospital, No. 270, Tianhui Road, Jinniu District, Chengdu, 610083 People’s Republic of China

**Keywords:** HAGOS, Total hip arthroplasty, Reliability, Validity, Responsiveness, Quality of life

## Abstract

**Background:**

To translate and cross-culturally adapt the Copenhagen Hip and Groin Outcome Score (HAGOS) into a Simplified Chinese version (HAGOS-C) and evaluate the reliability, validity, and responsiveness of the HAGOS-C in total hip arthroplasty (THA) patients.

**Methods:**

The cross-cultural adaptation was performed according to the internationally recognized guidelines of the American Academy of Orthopaedic Surgeons Outcome Committee. A total of 192 participants were recruited in this study. The intra-class correlation coefficient (ICC) was used to determine reliability. Construct validity was analyzed by evaluating the correlations between HAGOS-C and EuroQoL 5-dimension (EQ-5D), as well as the short form (36) health survey (SF-36). Responsiveness of HAGOS-C was evaluated according to standard response means (SRM) and standard effect size (ES) between the first test and the third test (6 months after primary THA).

**Results:**

The original version of the HAGOS was well cross-culturally adapted and translated into Simplified Chinese. HAGOS-C was indicated to have excellent reliability (ICC = 0.748–0.936, Cronbach’s alpha = 0.787–0.886). Moderate to substantial correlations between subscales of HAGOS-C and EQ-5D (*r* = 0.544–0.751, *p* < 0.001), as well as physical function (*r* = 0.567–0.640, *p* < 0.001), role physical (*r* = 0.570–0.613, *p* < 0.001), bodily pain (*r* = 0.467–0.604, *p* < 0.001), and general health (*r* = 0.387–0.432, *p* < 0.001) subscales of SF-36, were observed. The ES of 0.805–1.100 and SRM of 1.408–2.067 revealed high responsiveness of HAGOS-C.

**Conclusions:**

HAGOS-C was demonstrated to have excellent acceptability, reliability, validity, and responsiveness in THA, which could be recommended for patients in mainland China.

## Background

Total hip arthroplasty (THA) has demonstrated among the most successful operations in medicine [1, 2] and proven effective in patients with hip diseases [3, 4], which has a profound impact on health-related quality of life (HRQoL) [5]. For a better understanding of patient disorder severity and more appropriate therapeutic approach [6], a large body of patient-based HRQoL questionnaires have been developed [7], such as Copenhagen Hip and Groin Outcome Score (HAGOS) [8]. This need has become more essential with the growing number of multicenter studies among different countries and cultures [7], which provide more statistical power of evidence-based trials [9]. When one reliable, valid questionnaire is used in populations with different cultures, it is necessary to test the psychometric properties of the questionnaire rather than simply translating the content to avoid bias due to cultural variety [10, 11].

The HAGOS, published in 2012, consists of 37 items in six subscales: symptoms (7 items), pain (10 items), function in daily living (5 items), function in sport and recreation (8 items), participation in physical activities (2 items), and hip- and/or groin-related quality of life (5 items) [8]. A Danish, English, Swedish, and Dutch version of HAGOS was distinguished in good reliability and validity [8, 12, 13] and has been widely used in assessing patients with hip disorders. Chinese is the language spoken by the largest population in the world, and China has one of the largest population of patients performed with total joint arthroplasty and arthroscopy. However, there is no HAGOS in Chinese version for this population so far. Besides, as a scale evaluating hip problems, no study has been performed to validate HAGOS in arthroplasty patients.

Considering the cultural gap and social environment between China and western countries, the purpose of this study was to translate, adapt the original version of HAGOS into a Simplified Chinese version (HAGOS-C) cross-culturally, and evaluate the reliability, validity, and responsiveness of HAGOS-C in native Chinese-speaking patients who underwent THA.

## Methods

### Translation and cross-cultural adaptation

The steps of translation and trans-cultural adaptation were followed by previous guidelines in five steps [7, 14]. Forward translation—two bilingual translators translated the scale from English to simplified Chinese independently. One of the translators was an orthopedic surgeon in the author’s hospital; the other one was a professional translator without medical background. Synthesis of the translation—two translators and other researchers unified contradictions regarding language expression and cultural difference after a consensus meeting and obtained the first HAGOS-C. Backward translation—two native English speakers with fluent English and blind to the previous original English version of HAGOS independently translated the first HAGOS-C back into English version. Summarization of prefinal HAGOS-C—a consensus meeting with all researchers including four forward and backward translators was held to resolve all discrepancies, ambiguities, or any other verbal issues to reach a prefinal HAGOS-C. Determination of final HAGOS-C—researchers invited 20 patients to preliminarily test the prefinal version and collect feedback from them.

Eventually, all researchers involved in this study discussed issues in translation and developed the final HAGOS-C.

### Patients and data collection

From August 2015 to September 2017, 192 participants were recruited from patients suffering from developmental dysplasia of hip joint, osteonecrosis of the femoral head, or hip osteoarthritis for total hip arthroplasty in two hospitals of authors. The inclusion criteria were as follows: age > 18 years of age, literate native Chinese speakers, and patients diagnosed with diseases above that required THA. Participants were excluded for similar symptoms at contralateral limb; inflammatory joint diseases, such as ankylosing spondylitis and rheumatoid arthritis; hip operation history; history of spine surgery or any surgery in the recent 1 month; other diseases that limited patient sport or movement ability; other uncontrolled systematic disorders, such as diabetes mellitus, malignant tumor, or hepatitis. Participants who met the inclusion criteria and presented no item in the exclusion criteria were recruited in this study. The number of patients also needed to meet the standard proposed by Terwee et al. [15] that the study should include at least 50 patients for floor or ceiling effects, reliability, and validity analysis. All included participants were required to sign informed consent, and the study was approved by the clinical research Ethics Committees of hospitals of authors.

Patients should provide demographic data regarding gender, year of age, side of affected joint, and diagnosis at the first day approving to participate the study and then finished the HAGOS-C, EuroQoL 5-dimension (EQ-5D), and the short form (36) health survey (SF-36). All participants filled in the HAGOS-C for the second time 7–14 days later before surgery to assess its test-retest reliability and were contacted 6 months postoperatively to complete HAGOS-C to assess its responsiveness.

### Instruments

The Hip and Groin Outcome Score (HAGOS) is a disease-specific questionnaire for people suffering from hip and/or groin complaints [8]. It consists of 37 items in six subscales, symptoms (7 items), pain (10 items), function in daily living (ADL, 5 items), function in sport and recreation (sport/rec, 8 items), participation in physical activities (PA, 2 items), and hip- and/or groin-related quality of life (QoL, 5 items). In this questionnaire, all questions are answered from extreme symptoms to no symptoms, corresponding to 0 to 4 scores. Total points for each subscale are calculated according to the average score of all answered questions (eliminating missing value) and then multiplied by 25 into the centesimal system (0–100 scores). Higher scores refer to better outcome.

The EQ-5D is a self-reported questionnaire which consists of two pages, EQ-5D descriptive system, and EQ visual analog scale (EQ-VAS). EQ-5D descriptive system records the level of problems in five dimensions including mobility, self-care, usual activities, pain/discomfort, and anxiety/depression, and EQ-VAS records the respondent’s self-rated health on a visual analog scale where the endpoints are labeled “best imaginable health state” and “worst imaginable health state” [16]. SF-36 is a questionnaire assessing the general quality of life. It is composed of 36 items in eight subscales to evaluate the patient’s general condition. Scores for each subscale range from 0 (poor) to 100 (good) [17]. Both of the scales above have been translated into Chinese and proven good reliability and validity [18–20].

### Psychometric assessments and statistical analysis

To assess the acceptability of HAGOS-C, patients were asked for the difficulties encountered. Statistical analysis for score distribution was performed. Floor and ceiling effects were defined as being present if more than 15% of patients reported lowest (0) or highest (100) possible scores [8, 21].

Reliability was examined in terms of test-retest reliability and internal consistency. The test-retest reliability was tested by comparing outcomes when the same patient without changes in health answered HAGOS-C at two separated situations with proper duration interval. It was evaluated by the intra-class correlation coefficient (ICC), which derived from a two-way analysis of variance in a random effect model. ICC > 0.8 and > 0.9 were considered as good and excellent reliability [22]. Bland-Altman plots were carried out to estimate systematic bias between the two measures [23]. Meanwhile, Cronbach’s alpha was used to assess the internal consistency of the questionnaire, and > 0.7, 0.8, and 0.9 was considered as acceptable, good, and excellent internal consistency, respectively [15].

Validity tests for HAGOS-C included content validity and construct validity. To assess content validity, one rehabilitation therapist and three orthopedists were invited to analyze the correlation between content in each item and state of disease. Good construct validity meant that the questionnaire correlated well with measures of the same construct (convergent validity) and correlated poorly with measures of different constructs (divergent or discriminant validity) [24]. On account of this theory, we assumed that the score of HAGOS-C should be in accordance with EQ-VAS and disease-related subscales of EQ-5D and SF-36, but not with other subscales of EQ-5D and SF-36. Under such hypothesis, we calculated Pearson correlation coefficient ® between HAGOS-C and EQ-VAS and subscales of EQ-5D and SF-36. Then, the construct validity for HAGOS-C was evaluated by comparing how data conformed to the calculated correlations, judged as poor (*r* = 0–0.2), fair (*r* = 0.2–0.4), moderate (*r* = 0.4–0.6), substantial (*r* = 0.6–0.8), or almost perfect (*r* = 0.8–1.0) [24].

The responsiveness of HAGOS-C was evaluated according to standard response means (SRM) and standard effect size (ES) between the first test and the third test (6 months after primary THA). SRM represented the mean change score divided by the SD of the change score. The ES was calculated as the mean change in score divided by the SD of the baseline score [25]. SRM was considered large if larger than 0.80, moderate if between 0.50 and 0.79, and small if between 0.2 and 0.49. For ES, a value of 0.80 or higher was considered as high responsiveness.

Statistical Package for the Social Sciences, version 20.0 (SPSS, Chicago, IL) was used to analyze data. *p* values of 0.05 or less were considered significant.

## Results

### Participants

From August 2015 to September 2017, 238 patients were invited to participate in our study, and 192 of them (80.7%) agreed to participate in the study. All patients completed two rounds of instruments without withdrawn cases, in which 158 (82.3% of patients included) finished the third round of instrument assessing responsiveness. Detailed demographic and clinical characteristics of participants were listed in Table 1.

**Table 1:** Demographic and clinical characteristics of participants

Characteristics	Number or mean ± SD
Age (years)	64.1 ± 12.7
Range	28–88
Gender	Total (*N* = 192)
Female	112 (58.3%)
Male	80 (41.7%)
Side	
Right	85 (44.3%)
Left	107 (55.7%)
Diagnosis	
Developmental dysplasia of hip joint	87 (45.3%)
Osteonecrosis of the femoral head	73 (38.0%)
Hip osteoarthritis	32 (16.7%)

### Translation and cross-cultural adaptation process

There were no major problems in the forward and back translations of HAGOS. However, “vacuuming” in the item A5 listed in the original English version of HAGOS were less popular among Chinese and were adapted cross-culturally into “sweeping floors.” After the adaptation, no special issue was raised by the participants in the prefinal test. In consequence, the final version of HAGOS-C could be used to evaluate the patients’ condition in further research.

### Acceptability and score distribution

In formal investigation, no participants complained that any content was too difficult to understand at the first time of completing HAGOS-C. The answer rate was 100%.

Neither ceiling effect nor floor effect was significant in all subscales of HAGOS-C, except floor effect for the subscale PA (20.3%) (Table 2).

**Table 2:** Score distribution and floor-ceiling effects of the subscales of HAGOS-C

Subscale	Mean ± SD	Observed range	Theoretical range	Floor effect (%)*	Ceiling effect (%)*
Symptoms	44.9 ± 16.9	3.6–82.1	0–100	0	0
Pain	39.2 ± 15.8	0–87.5	0–100	2.1	0
ADL	49.3 ± 23.9	0–100	0–100	0.5	1.6
Sport/rec	36.5 ± 19.3	0–90.6	0–100	3.1	0
PAsss	26.3 ± 24.0	0–100	0–100	20.3	1.0
QoL	34.1 ± 21.2	0–95	0–100	6.3	0

*Percentage of patients with the worst (floor effect) and the best (ceiling effect) condition

*HAGOS-C* Chinese version of the Copenhagen Hip and Groin Outcome Score, *ADL* physical function in daily living, *sport/rec* physical function in sport and recreation, *PA* participation in physical activities, *QoL* hip and or/groin-related quality of life

### Reliability

**Fig. 1:**
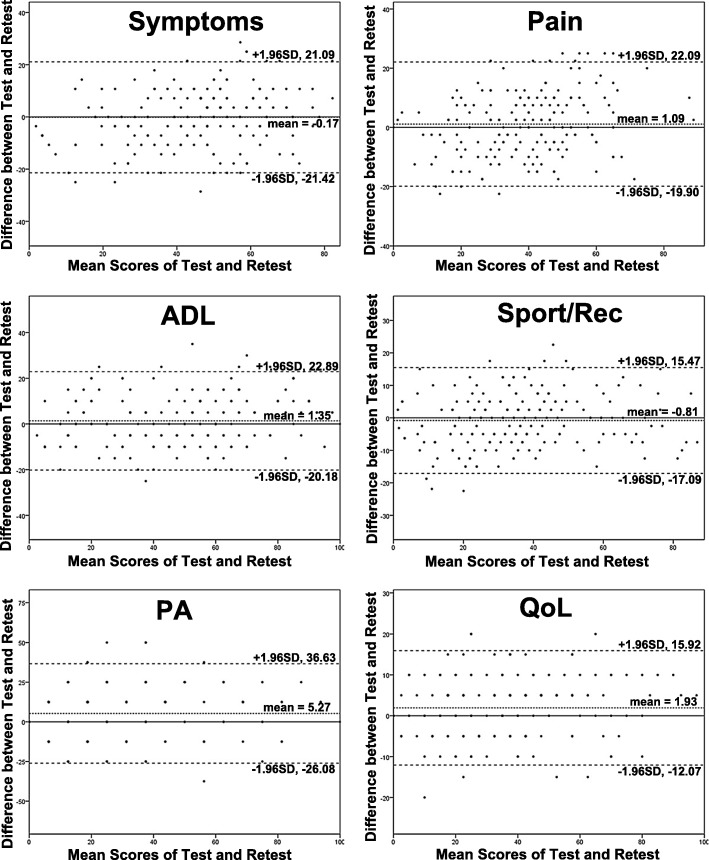
The Bland-Altman plot for test-retest agreement of HAGOS. The differences between scores for HAGOS from first two test sessions were plotted against the mean of the test and retest. The line indicates mean difference value of the two sessions and the 95% (mean ± 1.96 standard deviation) limits of agreement

Mean scores of each subscale in the retest was comparable with the first test (Table 3). ICCs ranged from 0.748 to 0.936, demonstrating good or excellent test-retest reliability of HAGOS-C. Bland-Altman plots for the two measures revealed no systematic error (Fig. 1), which suggested good test-retest accordance and reproducibility of HAGOS-C [23]. Cronbach’s alpha coefficient was calculated for each subscale ranging from 0.787 to 0.886, indicating a high internal consistency.

**Table 3:** Reliability and responsiveness of the HAGOS-C

Subscale	1st test (mean ± SD)	2nd test (mean ± SD)	3rd test (mean ± SD)	ICC (CI range)	ES	SRM	Cronbach’s alpha
Symptoms	45.3 ± 16.9	45.1 ± 19.5	64.3 ± 16.2	0.824 (0.773–0.865)	1.100	1.868	0.812
Pain	38.8 ± 15.8	39.9 ± 18.5	56.2 ± 13.7	0.806 (0.750–0.850)	1.096	1.949	0.787
ADL	47.6 ± 23.9	49.0 ± 25.8	69.6 ± 20.2	0.902 (0.872–0.926)	0.849	2.067	0.886
Sport/rec	36.5 ± 19.3	35.6 ± 20.4	57.2 ± 18.6	0.913 (0.886–0.934)	1.057	1.797	0.858
PA	27.5 ± 24.0	32.7 ± 25.7	48.4 ± 24.2	0.793 (0.734–0.840)	0.805	1.408	0.866
QoL	33.8 ± 21.2	35.8 ± 22.2	54.5 ± 21.0	0.946 (0.929–0.959)	0.944	1.644	0.873

*The 1st test was conducted at the beginning of this research (192 patients), the 2nd test was conducted 1 week later to calculate the test-retest reliability (ICC) of the HAGOS-C (192 patients), and the 3rd test was conducted 6 months later to calculate the responsiveness (ES, SRM) of the HAGOS-C (158 patients)

*ICC* intra-class correlation coefficient, *ES* effect size, *SRM* standardized response mean, *CI* 95% confidence interval, *HAGOS-C* Chinese version of the Copenhagen Hip and Groin Outcome Score

### Validity

According to the evaluation of rehabilitation expert and orthopedic experts, the content validity was good in HAGOS-C and the information derived from all items was adequate to assess the function of included patients.

Table 4 lists the data of construct validity of HAGOS-C. All subscales of the HAGOS-C showed significant correlations with the EQ-5D-S total score and the EQ-5D-S VAS score (Table 4). When comparing HAGOS-C with SF-36, correlation coefficients for subscales of physical function (*r* = 0.567–0.640, *p* < 0.001), role physical (*r* = 0.570–0.613, *p* < 0.001), bodily pain (*r* = 0.467–0.604, *p* < 0.001), and general health (*r* = 0.387–0.432, *p* < 0.001) were moderate to substantial; meanwhile, this correlation was just weak to fair for vitality (*r* = 0.195–0.256, *p* = 0.001–0.007), social function (*r* = 0.240–0.313, *p* < 0.001), role emotional (*r* = 0.141–0.247, *p* = 0.001–0.051), and mental health (*r* = 0.168–0.276, *p* = 0.001–0.020), which consistently matches our hypothesis.

**Table 4:** Construct validity of the HAGOS-C

Correlation coefficient *r*_*s*_ (*p* value)*	HAGOS-C subscales					
	Symptoms	Pain	ADL	Sport/rec	PA	QoL
EQ-5D						
Total score	0.751 (< 0.001)	0.637 (< 0.001)	0.605 (< 0.001)	0.637 (< 0.001)	0.544 (< 0.001)	0.625 (< 0.001)
Health status (VAS)	0.671 (< 0.001)	0.534 (< 0.001)	0.495 (< 0.001)	0.523 (< 0.001)	0.513 (< 0.001)	0.523 (< 0.001)
SF-36 subscales						
Physical function	0.601 (< 0.001)	0.568 (< 0.001)	0.640 (< 0.001)	0.602 (< 0.001)	0.567 (< 0.001)	0.593 (< 0.001)
Role-physical	0.589 (< 0.001)	0.598 (< 0.001)	0.579 (< 0.001)	0.613 (< 0.001)	0.573 (< 0.001)	0.570 (< 0.001)
Bodily pain	0.567 (< 0.001)	0.604 (< 0.001)	0.490 (< 0.001)	0.557 (< 0.001)	0.467 (< 0.001)	0.541 (< 0.001)
General health	0.432 (< 0.001)	0.391 (< 0.001)	0.407 (< 0.001)	0.406 (< 0.001)	0.406 (< 0.001)	0.387 (< 0.001)
Vitality	0.195 (0.007)	0.243 (0.001)	0.218 (0.002)	0.232 (0.001)	0.256 (< 0.001)	0.221 (0.002)
Social function	0.282 (< 0.001)	0.278 (< 0.001)	0.240 (0.001)	0.286 (< 0.001)	0.313 (< 0.001)	0.282 (< 0.001)
Role-emotional	0.168 (0.020)	0.141 (0.051)	0.169 (0.019)	0.178 (0.014)	0.247 (0.001)	0.180 (0.012)
Mental health	0.177 (0.014)	0.203 (0.005)	0.168 (0.020)	0.195 (0.007)	0.276 (< 0.001)	0.198 (0.006)

*Calculated by the Pearson correlation coefficient of the HAGOS-C with EQ-5D and SF-36

*HAGOS-C* Chinese version of the Copenhagen Hip and Groin Outcome Score, *EQ-5D* EuroQol-5 dimensions, *SF-36* short form 36, *ADL* physical function in daily living, *sport/rec* physical function in sport and recreation, *PA* participation in physical activities, *QoL* hip and or/groin-related quality of life

### Responsiveness

To assess responsiveness, 158 participants completed the third round of instrument approximately 6 months after primary THA. ES ranged from 0.805 to 1.100, and SRM ranged from 1.408 to 2.067 (Table 3). Both ES and SRM were greater than 0.80, indicating high responsiveness for all subscales.

## Discussion

In this study, the English version of HAGOS was successfully translated and cross-culturally adapted into Simplified Chinese. The HAGOS-C had good reliability, validity, and responsiveness in evaluating patients who underwent THA in mainland China.

HRQoL questionnaires are very important and valuable in the quantification of patients’ function and data analysis among studies. Nowadays, with the invigorating strategy through science, technology and education, and greater science and technology input in China, the number of papers annually published in China is the second largest all over the world [24, 26, 27]. Therefore, valid questionnaires are urgently needed to support this huge amount of clinical research.

In the process of translation and adaptation, authors strictly followed the standardized procedure listed in the literature. In item A5, “vacuuming” written in the original English version of HAGOS were less popular among Chinese and were adapted cross-culturally into “sweeping floors”. Interestingly, with the popularity of price-friendly “sweeping and mopping robot” in China, better examples listed in A5 in HAGOS-C might be explored to substitute “scrubbing and sweeping floors”.

A floor effect of 20.8% was observed in the subscale of PA in HAGOS-C, which was also detected in literature before [8, 12, 13]. This relative high floor effect might be due to the following reasons. Firstly, there are only two items listed in this subscale, which makes it easy to choose both of the items with the lowest score. Besides, some of the patients who underwent THA suffered from end-stage hip diseases, which restricted patients from participation in physical activities naturally.

In our study, all subscales of HAGOS-C showed very good internal consistency (Cronbach’s alpha = 0.787–0.886) and test-retest reliability (ICC = 0.793–0.946). The results above were basically in agreement with the data reported by Thorborg et al. (Danish HAGOS), Thomeé et al. (Swedish HAGOS), and Brans et al. (Dutch HAGOS) [8, 12, 13]. The ICC for the QoL subscale (ICC = 0.946) is the highest among all subscales, which might due to the fact that quality of life for patients changed with least possibility in the duration interval of 1 to 2 weeks among the perspectives assessed in HAGOS-C.

The correlation between the subscales of HAGOS-C and EQ-5D total score, EQ-VAS, as well as SF-36 subscales, was in accordance with our hypothesis. Almost all correlations between HAGOS-C subscales and EQ-5D total score, EQ-VAS, as well as SF-36 subscales, were significant, except the correlation between pain subscale of HAGOS-C and role-emotional subscale of SF-36. However, the *r* value for these correlations varied a lot. In our study, HAGOS-C subscales correlated better with the EQ-5D total score, EQ-VAS, and physical function, role physical, and bodily pain subscales of SF-36, whereas these correlations were weaker between HAGOS-C subscales and vitality, social function, role-emotional, and mental health subscales of SF-36. One possible reason might be that HAGOS-C was designed for the evaluation of function and symptoms in the hip and groin region, and vitality, social function, role-emotional, and mental health subscales of SF-36 indicated psychological or social state of patients, which could be affected by many factors other than physical situation and symptoms comparing with other scales of high correlation with HAGOS-C. Interestingly, the correlation between symptoms, pain, and sport/rec subscales of HAGOS-C and EQ-5D and physical function, role-physical, bodily pain, and general health subscales of SF-36 were the slightly higher other subscales of HAGOS-C. Likewise, this might contribute to the fact that symptoms, pain, and sport/rec subscales of HAGOS-C indicated direct symptoms of patients, which were affected more by the disease itself with less interference of other matters. All of these suggested satisfied divergent or discriminant validity for HAGOS-C in THA patients.

The responsiveness was tested to detect changes between the preoperative and 6-month postoperative conditions. As our hypothesis, SRM and ES were defined as large after 6 months of postoperative rehabilitation. This outcome is similar to some part of other versions of the HAGOS. The ESs for the change in the score on the Danish version of HAGOS were − 1.29 to − 0.60, 0.01 to 0.19, and 0.77 to 1.78, in “much worse” and “worse” group, “somewhat worse” and “not changed” and “somewhat better,” and “much better” and “better” group, respectively[8]. Analogously, ESs on the Swedish version were − 0.44 to − 0.19, 0.23 to 0.54, and 1.07 to 1.87 in 20 points lower, ± 20 points, and 20 points higher of global perceived effect group, respectively [13]. The ESs in our study was much larger than the first two groups in both of the studies above, but comparable with the third group in these two studies. In the original Danish study, authors included patients seeking medical care presenting with hip and/or groin who had received treatment for the symptom, and in the cross-cultural study on Swedish, patients requiring hip arthroscopy for femoroacetabular impingement were investigated. Meanwhile, only patients who underwent THA were included in our study. Under the circumstances, the patients’ symptom severity in both of the studies above was milder than our study. As we know, THA has demonstrated among the most successful operations in medicine [1, 2], which has been proven effective in patients with hip diseases [3, 4]; so, it is reasonable that larger ESs were shown among patients who underwent THA.

There are several limitations to our study. First, the sample was limited in size and may not fully represent the Chinese population. Second, although Simplified Chinese is the official language in China, China is a country with multiple nationalities, most of which have their own language. Thus, the problem of national cultural differences should be noted. Finally, patients with symptoms in the hip and/or groin region who were not performed with THA were not evaluated, which could be carried out in future studies.

## Conclusion

The HAGOS was successfully translated and cross-culturally adapted into Simplified Chinese. The HAGOS-C had good reliability, validity, and responsiveness in evaluating patients who underwent THA in mainland China.

## Abbreviations

ADL: Function in daily living
EQ-5D: EuroQoL 5-dimension
EQ-VAS: EQ visual analog scale
ES: Effect size
HAGOS: Copenhagen Hip and Groin Outcome Score
HAGOS-C: Simplified Chinese version of Copenhagen Hip and Groin Outcome Score
HRQoL: Health-related quality of life
ICC: Intra-class correlation coefficient
PA: Participation in physical activities
QoL: Groin-related quality of life
SF-36: Short form (36) health survey
Sport/rec: Function in sport and recreation
SRM: Standard response means
THA: Total hip arthroplasty
